# Crossing exceptional points without phase transition

**DOI:** 10.1038/s41598-018-36701-9

**Published:** 2019-01-15

**Authors:** Qi Zhong, Ramy El-Ganainy

**Affiliations:** Department of Physics and Henes Center for Quantum Phenomena, Michigan Technological University, Houghton, MI 49931 USA

## Abstract

We show that the theoretical framework linking exceptional points (EPs) to phase transitions in parity-time (PT) symmetric Hamiltonians is incomplete. Particularly, we demonstrate that the application of the squaring operator to a *Jx* PT lattice dramatically alter the topology of its Riemann surface, eventually resulting in a system that can cross an EP without undergoing a symmetry breaking. We elucidate on these rather surprising results by invoking the notion of phase diagrams in higher dimensional parameter space. Within this perspective, the canonical PT symmetry breaking paradigm arises only along certainprojections of the Riemann surface in the parameter space.

## Introduction

Exceptional points (EPs) are peculiar singularities associated with multivalued complex function^1^. They also arise as special degeneracies in the spectra of parity-time- (PT-) symmetric (and in general non-Hermitian) Hamiltonians at which the eigenvalues and the corresponding eigenvectors become identical^2,3^, thus signaling a collapse of the eigenspace dimensionality, which in turn gives rise to a host of intriguing effects in the vicinity of these singularities^4,5^. One particular characteristic feature that has been studied thoroughly in literature is phase transitions across EPs in PT-symmetric arrangements^6^. When the symmetry of the eigenvectors are studied as a function of one parameter of the PT Hamiltonian, one finds that on one side of the EP, the eigenvectors respect PT symmetry (i.e. they commute with the PT-symmetric operators) whereas on the other side of the EP, they violate the PT symmetry (in fact applying PT operator to one eigenvector yields another eigenvector). This phase transition between the PT phase and broken PT (BPT) phase, which is also known as PT spontaneous symmetry breaking, is accompanied by complex eigenvalue bifurcation. This behavior has been experimentally demonstrated in various physical platforms in optics^7–10^, electronics^11^ and acoustics^12^. For more information, see refs^13,14^.

In light of these intense theoretical and experimental activities, it is perhaps not surprising that EPs are always associated PT phase transitions. What is surprising though, is the lack of any rigorous mathematical proof for this statement. In this work, we show that this is not a coincidence, and that this widely accepted picture of PT phase transition is in fact incomplete.

Before we proceed, we briefly review the archetypal discrete PT-symmetric Hamiltonian, which was the subject of detailed investigation in several studies^7,9,10,15,16^. It consists of two coupled elements, having coupling coefficient *κ* and balanced gain/loss profile characterized by the non-Hermitian parameter *γ*. This Hamiltonian respects PT symmetry in the *κ* − *γ* plane. However, as *γ* is varied from *γ* < *κ* to *γ* > *κ*, the associated eigenvectors undergo a spontaneous symmetry breaking from the PT phase to the BPT phase^13,14^. The transition point separating these two phases (*γ* = *κ*) is an EP. This behavior, which we call canonical PT phase transition, has been reported in more complex discrete and continuous systems. This in turn led to the common belief that crossing EPs along straight lines and PT phase transitions are inseparable notions.

In this work, we show that this is not the whole story and that this picture is indeed incomplete. To do so, we use the squaring operator to construct a simple Hamiltonian that violates the canonical PT phase transition in the following sense: as one parameter is varied continuously and monotonically along a straight line, the system crosses an EP without any PT symmetry breaking. As we will shortly see, this is an outcome of the non-trivial topological features incurred on the Riemann surface by the squaring operation. For more detailed discussion on how square and square root operations can give rise to altogether new topological structures, see ref.^17^.

To this end, consider the following family of PT Hamiltonians *H*_*M*_ (which can be generated by using the recursive bosonic algebra method^18^) whose matrix elements are given by:

1 $${H}_{M}(n,l)=i2n\gamma {\delta }_{n,l}+\kappa {g}_{n+1}{\delta }_{n,l-1}+\kappa {g}_{n}{\delta }_{n,l+1},$$

where *γ* and *κ* are the gain (or loss) and coupling coefficients, respectively and ${g}_{n}=\sqrt{(N+n)(N-n+\mathrm{1)}}$; with *n*, *l* = −*N*, −*N* + 1, …‥, *N* − 1, *N* and *M* = 2*N*. Note that the value of *N* can be integer or half-integer.

**Figure 1:**
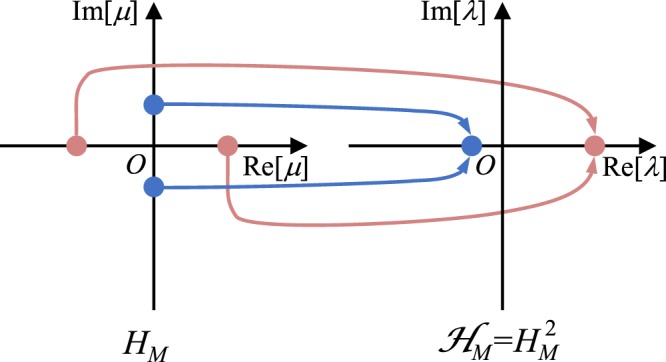
Illustration of the spectral properties of *H*_*M*_ and ${ {\mathcal H} }_{M}={H}_{M}^{2}$, as discussed in details in the text.

**Figure 2:**
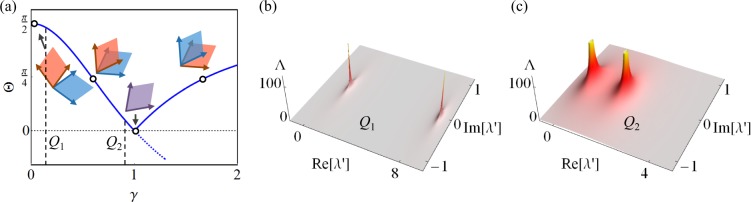
(**a**) The Hermitian angle Θ between the two planes spanned by the degenrate eigenvectors of that Hamiltonian ${ {\mathcal H} }_{3}$ as a function of the non-Hermitian parameter *γ*. At the point *γ* = 1, the two planes are parallel, indicating a reduction of the eigenspace dimensionality. (**b**) and (**c**) depict the parameter ${\rm{\Lambda }}=\Vert {({ {\mathcal H} }_{3}-\lambda ^{\prime} I)}^{-1}\Vert$ as a function of the complex parameter *λ*′. Close to the point *γ* = 1, the system exhibits sensitivity to perturbation as indicated by the large values of Λ over a wider area in the *λ*′ plane (see SI for discussion on pseudospectrum). These results confirm that *γ* = 1 is indeed an EP of ${ {\mathcal H} }_{M}$.

The eigenvalues of *H*_*M*_ are given by ${\mu }_{M,m}=(M-2m)\sqrt{{\kappa }^{2}-{\gamma }^{2}}$ where *m* = 0, 1, 2, ‥‥, *M*^18^; and they feature higher order EPs^19–22^ at *γ* = *κ* with a phase transition across this point from PT phase (*γ* > *κ*) to the BPT phase (*γ* > *κ*)^18,23^. The crucial observation here is that the eigenvalues are pure real (imaginary) in the PT (BPT) phase. Let us now consider a new Hamiltonian ${ {\mathcal H} }_{M}={H}_{M}^{2}$. First we note that ${ {\mathcal H} }_{M}$ respects PT symmetry since $PT{ {\mathcal H} }_{M}{(PT)}^{-1}=PT{H}_{M}{(PT)}^{-1}PT{H}_{M}{(PT)}^{-1}={ {\mathcal H} }_{M}$. Furthermore, The eigenvalues of ${\mathcal H}$ are given by *λ* = *μ*^2^. The eigenspectrum of ${ {\mathcal H} }_{M}$ is thus composed of degenerate subspaces with positive real eigenvalues when *γ* < *κ* and negative real eigenvalues when *γ* > *κ*. A schematic representation of how this degeneracy arise is shown in Fig. 1. Clearly, the spectrum of ${ {\mathcal H} }_{M}$ does not undergo complex eigenvalue bifurcation as the parameter *γ* is swept across a straight line that passes through the point *γ* = *κ*. Additionally, an important consequence of the two-fold degeneracy of the spectrum of ${ {\mathcal H} }_{M}$ is that, it is always possible to construct PT symmetric eigenstates for any *γ* and *κ* which is not the case for *H*_*M*_ (see Supplementary Note A for more detailed discussion). It thus appears that the point *γ* = *κ* is not associated with phase transition. Naturally, one would then ask if it is an EP. In order to check this and without in loss of generality, we consider the case where *M* = 3 (the Hamiltonian ${ {\mathcal H} }_{3}$ has dimensions 4×4) and we set *κ* = 1. In general the Hamiltonian ${ {\mathcal H} }_{3}$ has four different eigenstates forming two degenerate subspaces. Figure 2(a) depicts the Hermitian angle (Θ) between the two planes consisting of the degenerate eigenvectors (see Supplementary Note B for the definition of Hermitian angle between two planes). One indeed sees that at *γ* = 1, the two planes are identical (Θ = 0), which indicates a collapse of the eigenspace dimensionality as would be expected at an EP. Interestingly however, at this point ${ {\mathcal H} }_{3}$ exhibit two different eigenvectors whereas *H*_3_ has only one. We elaborate more on this feature in Supplementary Note A. Figure 2(b) and (c) plot the values of ${\rm{\Lambda }}=\Vert {({ {\mathcal H} }_{3}-\lambda ^{\prime} I)}^{-1}\Vert$ as a function of the parameter *λ*′ for two different cases when *γ* = 0.1 (away from the EP) and *γ* = 0.9 (close to the EP). The spread of the high values of Λ in the *λ*′ plane indicates the system sensitivity to perturbations (see Supplementary Note C for detailed definition of pseudospectrum method). As can be inferred from the figure, this is indeed the case in the vicinity of point *γ* = 1. Based on the above analysis, we indeed conclude that, as the Hamiltonian ${ {\mathcal H} }_{3}$ is swept across the straight line passing through the point *γ* = 1, it crosses an EP without experiencing a phase transition. The above example thus presents a very interesting scenario that demonstrates the possibility of violating the canonical PT symmetry breaking (in Supplementary Note D we show that this example is not unique).

**Figure 3:**
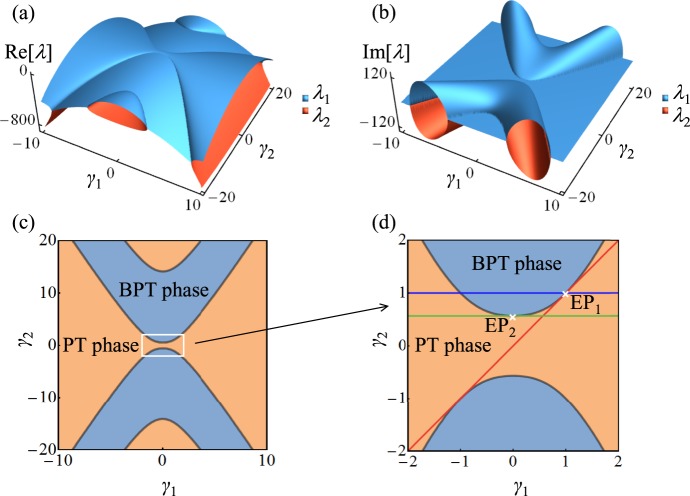
(**a**) and (**b**) Riemann surfaces for real and imaginary components of the spectrum of ${ {\mathcal H} }_{3}$ are depicted as a function of the two parameters *γ*_1,2_ (see text for details). (**c**) The phase diagram associated with (**a**) and (**b**). (**d**) Magnified view of the central part of (**c**). The red line represents the trajectory *γ*_1_ = *γ*_2_ corresponding to Fig. 2(a) and crosses EP_1_ without phase transition. Other trajectories such as that shown by the green line and cross EP_2_ can also demonstrate similar behavior. On the other hand, the horizontal trajectory having *γ*_2_ = 1 (blue line) is associated with phase transition.

In order to better understand these results within a general framework that encompass all the possible different situations, we invoke the notion of phase diagrams. In statistical mechanics and nonlinear dynamics, phase diagrams are used to classify the system’s behavior into different phases as a function of some external parameters. In the context of our discussion of ${ {\mathcal H} }_{3}$, one should in general study the classification of the eigenstates as a function of 32 different parameters (16 complex matrix entries). Fortunately, we can gain an insight into the behavior of ${ {\mathcal H} }_{3}$ by considering a low dimensional projection of this higher dimensional parameter space. Here we do so by fixing *κ* and de-correlate some of the other parameters by allowing ${ {\mathcal H} }_{3}\mathrm{(1},\mathrm{1)}$ and ${ {\mathcal H} }_{3}\mathrm{(4},\mathrm{4)}$ to vary as a function of *γ*_1_ while the rest of the matrix elements vary with *γ*_2_. This choice provides a 2D projection of the phase diagram while at the same time guarantees that ${ {\mathcal H} }_{3}$ still respect PT symmetry. Figure 3(a) and (b) depict the Riemann surfaces for the real and imaginary components of the eigenvalues of ${ {\mathcal H} }_{3}$ in the *γ*_1_ – *γ*_2_ plane, where one can identify the distinct PT phases. Figure 3(c) plots the phase diagram as extracted from Fig. 3(a). Figure 3(d) presents a more detailed blow up of the area surrounded by the rectangle in (c). Figure 3(d) clearly demonstrates the different phases are separated by curved lines of EPs. As one varies one or more of the system’s parameter, the behavior can be very different depending on the trajectory taken in the parameter space. For example, no phase transition is observed if *γ*_1_ is varied while *γ*_2_ = 0. On the other hand, fixing *γ*_2_ = 1 and sweeping *γ*_1_ from negative to positive values will lead to a PT-BPT phase transition followed by a BPT-PT transition (blue line). One can also fix ${\gamma }_{2}=\sqrt{100-12\sqrt{69}}\approx 0.566$ to a value that guarantees that the line swept by varying *γ*_1_ will just touch one EP without any phase transition (green line touching EP_2_). The particular case we discussed earlier for ${ {\mathcal H} }_{3}$ corresponds to the line *γ*_1_ = *γ*_2_ which also touches the boundary at one EP (red line crossing EP_1_) without any phase transition. The concept of higher dimensional phase diagram thus provides a unified umbrella to treat all the rather special cases of phase transition, reverse phase transition, EP without phase transition, no EP and no phase transition as well as multiple phase transitions. This generalized perspective is very important to complex non-Hermitian systems and design next generation experiments.

As a side note, we emphasize that the exceptional lines separating the different phases in Fig. 3 curves in the parameter space, which are very different from previous studies that demonstrated exceptional lines in the Fourier space^24,25^.

In summary, we have revisited the concept of PT phase transition across EPs and demonstrated that, contrary to the common belief, crossing an EP along straight lines (the case of curved trajectories is rather trivial) in the parameter space can take place without PT spontaneous symmetry breaking. We have explained these results by introducing the concept of PT phase diagram and its different projections in the parameter space that characterize a PT symmetric Hamiltonian. Our work provides a new twist and a deeper understanding for the physics of non-Hermitian systems near EPs, with potential implication in various fields such as photonics^14^, acoustics^12,26^ and electronics^11,27,28^. Particularly, the recent important work on driving Floquet PT symmetric systems offer a natural platform for confirming our predictions experimentally^29^.

**Figure 4:**
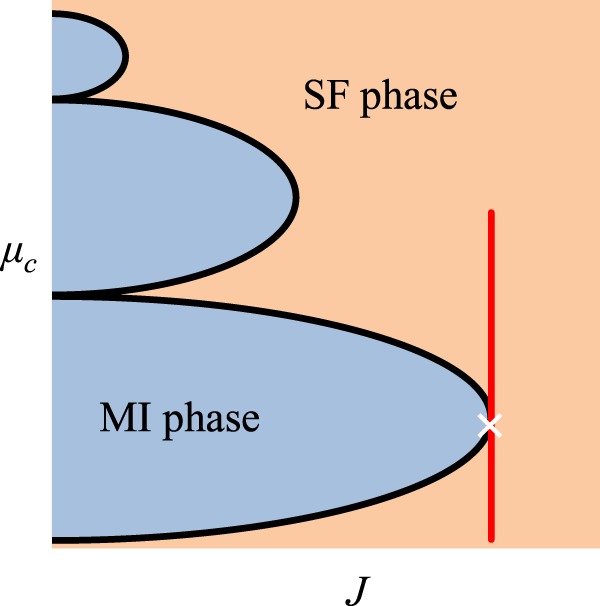
Generic phase diagram of the Bose-Hubbard model in a one dimensional lattice as a function of the hopping parameter *J* and the chemical potential *μ*_*c*_. A trajectory along the red line can cross the critical point without any transition from the superfluid (SF) phase to the Mott insulator (MI) phase.

Our results also raise interesting questions about the evolution along closed loops^30–37^ in these higher dimensional parameter spaces, which we plane to investigate elsewhere. Finally it is instructive to compare the behavior discovered here in this work with other systems studied in condensed matter physics. For example, by referring to the phase diagram of the quantum phase transition associated with the Bose-Hubbard Hamiltonian of interacting chain^38–40^ (Fig. 4), we can see that it is possible to choose a trajectory that crosses the critical point on the boundary between the superfluid (SF) and Mott insulator (MI) phases from the SF side without having a phase transition.

## Electronic supplementary material

Supplementary Information
