# Supplementary material for: Crossing exceptional points without phase transition
Source: Sci Rep. 2019 Jan 15;9:134. doi: 10.1038/s41598-018-36701-9 (PMC6333926; doi:10.1038/s41598-018-36701-9)
Supplement: Supplementary file 1 — Supplementary Information [file 41598_2018_36701_MOESM1_ESM.pdf]

Crossing exceptional points without phase transition: Supplementary information

Qi Zhong[1] **and Ramy El-Ganainy**[1,*]

[1]Department of Physics and Henes Center for Quantum Phenomena, Michigan Technological University, Houghton, MI, 49931, USA
[*]ganainy@mtu.edu

A. Eigenstates of $\mathscr{H}_M$

Here we discuss the different phases of the Hamiltonian $\mathscr{H}_M = H_M^2$ in more details. As discussed in the main text, both Hamiltonians respect PT symmetry. Furthermore, the spectrum of H_M is in the PT or BPT phases when $\gamma < \kappa$ and $\gamma > \kappa$, respectively. Given that in these two regimes, both H_M and $\mathscr{H}_M$ share the same eigenvectors, it may appear that $\mathscr{H}_M$ should also undergoes phase transition to the BPT as γ is increased beyond κ. As we have seen however, this is not the case and the eigenvalues of $\mathscr{H}_M$ remain real in all regimes. To reconcile these two observations, we recall that the spectrum of $\mathscr{H}_M$ consists of degenerate manifolds. Thus it is always to use superposition between any two degenerate two states to form a new bases that respect PT symmetry. For instance, consider two conjugate eigenstates of H_M, say $|\phi_{1,2}(\gamma)\rangle$ and having eigenvalues $\pm\mu$. For $\gamma < \kappa$, we will denote these eigenstates as $|\phi_{1,2}^{PT}\rangle$ since they satisfy PT symmetry: $PT\,|\phi_{1,2}^{PT}\rangle = |\phi_{1,2}^{PT}\rangle$. On the other hand, when $\gamma > \kappa$, we will denote them by $|\phi_{1,2}^{BPT}\rangle$ as they now break the PT symmetry: $PT\,|\phi_{1,2}^{BPT}\rangle = |\phi_{2,1}^{BPT}\rangle$. Note here that, without any loss of generality, we assumed a normalization of the eigenstates such that the action of the PT operator on them does not produce any scaling. When considering the phases of $\mathscr{H}_M$, both $|\phi_{1,2}(\gamma)\rangle$ are degenerate with an eigenvalue $\lambda = \mu^2$. Thus even when $\gamma > \kappa$, we can for new states $|\psi_\pm\rangle = e^{i(\pi/4 \mp \pi/4)}(|\phi_1^{BPT}\rangle \pm |\phi_2^{BPT}\rangle)$. It is straightforward to verify that both $|\psi_\pm\rangle$ satisfy PT symmetry with $PT\,|\psi_\pm\rangle = |\psi_\pm\rangle$. We note that this behavior of $\mathscr{H}_M$ is a peculiarity of the particular model but is not a necessary condition for crossing an EP without phase transitions. Another peculiarity of $\mathscr{H}_M$ is the possibility additional exceptional vectors (EVs) at the EP as compared with H_M. For example, while H_3 has only one EV, for $\mathscr{H}_3$ there are two independent EVs.

B. Hermitian angle between eigenplanes

In the main text, Fig. 3 (a), we plotted the Hermitian angle between two planes. Here we present the details of these calculations. Assume we have two planes defined by the vectors $v_{1,2}$ and $v_{3,4}$, respectively. One can then define the quantities $v_1 \wedge v_2$ and $v_3 \wedge v_4$ (called blades) and their inner product:

$$\langle v_1 \wedge v_2, v_3 \wedge v_4 \rangle = \begin{vmatrix} \langle v_1, v_3 \rangle & \langle v_1, v_4 \rangle \\ \langle v_2, v_3 \rangle & \langle v_2, v_4 \rangle \end{vmatrix}, \tag{1}$$

where $\langle , \rangle$ is the Hermitian inner product and $|...|$ is the determinant. Then the Hermitian angle Θ between these two planes is given by[1]:

$$\cos\Theta = \frac{|\langle v_1 \wedge v_2, v_3 \wedge v_4 \rangle|}{\sqrt{\langle v_1 \wedge v_2, v_1 \wedge v_2 \rangle \langle v_3 \wedge v_4, v_3 \wedge v_4 \rangle}}, \tag{2}$$

where $\Theta \in [0, \pi/2]$.

C. Pseudospectrum

In Figs. 3(b) and (c) in the main text, we use the notion os pseudospectrum[2] to study the system sensitivity close to the point $\gamma = 1$ in order to confirm its EP character. The ε-pseudospectrum of a matrix A, denoted as $\sigma_\varepsilon(A)$, is typically defined as:

$$\sigma_\varepsilon(A) = \{\lambda' \in \mathbb{C} : \lambda' \in \sigma(A+E) : ||E|| \leq \varepsilon\} \tag{3}$$

Here $\sigma(A)$ denotes the eigenvalue spectrum of A and $||...||$ is a matrix norm. Basically, it is a measure of how the eigenvalues of the original system vary in response for small perturbations. Near EPs, these variations are very large. This definition was used in connection with non-Hermitian photonics[3]. Another equivalent, more practical definition is:

$$\sigma_\varepsilon(A) = \{\lambda' \in \mathbb{C} : ||(A - \lambda' I)^{-1}|| \geq \frac{1}{\varepsilon}\} \tag{4}$$

where I is the unitary matrix. In the main text we plot the quantity $\Lambda \equiv ||(\mathscr{H} - \lambda' I)^{-1}||$ as a function of the complex parameter λ'. Near EPs, we expect a spread for the high values of Λ over a large area in the λ' plane.

D. Phase diagram of H_3

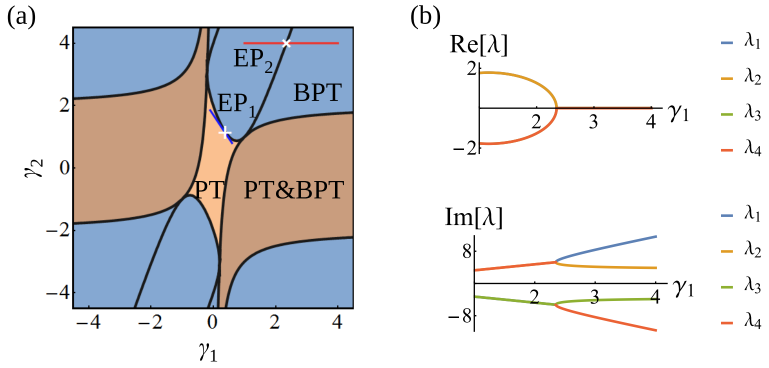

Figure S1. (a) The phase diagram of H_3 constructed by the method described in text. The parameter space is divided into PT, BPT and mixed PT&BPT phase by EP lines (black lines). In the PT regime, linear trajectories that cross an EP without phase transition can be constructed (see the trajectory represented by the blue line and crossing EP_1). The BPT domain exhibit exceptional lines that can be crossed without any change in the phase (see red line crossing EP_2). (b) present the details of the eigenvalue bifurcation along the crossing of EP_2. Clearly, the eigenvalues remain complex before and after the crossing.

In the main text, we used $\mathscr{H}_3 = H_3^2$ to exemplify the new behavior studied in this work, namely the existing of trajectories that can lead to a crossing of EPs without spontaneous symmetry breaking. Here we show that this particular case is not unique. As an example, let us consider H_3 itself without applying the squaring operator. Next, we allow $H_3(1,1)$ and $H_3(4,4)$ to vary as a function of γ_1 while $H_3(2,2)$ and $H_3(3,3)$ vary with γ_2. In the γ_1–γ_2 plane, H_3 always respects PT symmetry but its eigenstates can be in PT, BPT phase or a mixed PT&BPT (Only a subset of the eigenstates respect PT symmetry) phases, as shown in Fig. S1(a). The boundaries between the different phases consist of lines of EPs. Due to the curved nature of these exceptional lines, one can construct straight lines that cross EPs without any change in the phase. One of these trajectories in the PT phase is demonstrated on the figure (blue line segment crossing EP_1).

Interestingly, there are also exceptional lines extending in the BPT domain that do not separate any different phases. While the eigenvalues do bifurcate across these lines, they remain complex and the eigenvectors do not change their symmetry properties. For completeness, we present the spectral features as a trajectory crosses one of these lines (red line crossing EP_2) in Fig. S1(b).

References

1. Browne, J. *Grassmann Algebra. Exploring applications of extended vector algebra with Mathematica* (2001), web draft edn.

2. Trefethen, L., Embree, M. & Embree, M. *Spectra and Pseudospectra: The Behavior of Nonnormal Matrices and Operators* (Princeton University Press, 2005).

3. Makris, K. G., Ge, L. & Türeci, H. E. Anomalous transient amplification of waves in non-normal photonic media. *Phys. Rev. X* **4**, 041044 (2014).
